# Why modern family planning needs of women is not met in South Gondar Zone, Ethiopia?

**DOI:** 10.1371/journal.pgph.0000335

**Published:** 2022-06-07

**Authors:** Gojjam Eshetie Ewunetie, Mamo Dereje Alemu, Muluken Genetu Chanie

**Affiliations:** 1 Department of Medical Laboratory Sciences, Dembya Primary Hospital, North Gondar, Gondar, Ethiopia; 2 Health Systems Strengthening Directorate, Federal Ministry of Health, Addis Ababa, Ethiopia; 3 Department of Health Systems and Policy, School of Public Health, College of Medicine and Health Sciences, Wollo University, Dessie, Ethiopia; Pai Chai University, REPUBLIC OF KOREA

## Abstract

**Background:**

Family planning is critical for the health of women and their families and it can accelerate a country’s progress toward reducing poverty and achieving Sustainable Development Goals. Effective use of family planning methods helps couples achieve the desired number of children, contribute to improving maternal and child health which may help women avoid unwanted pregnancy, and reduce the risk factors for maternal and child deaths. Moreover, contraceptive prevalence and unmet need for family planning are key indicators for determining the level of improvements in access to reproductive health. So, this study aimed to identify the prevalence and associated factors of unmet need of modern family planning among reproductive-age women in the south Gondar zone.

**Methods:**

A community-based cross-sectional study design was conducted in the southern Gondar zone among 528 reproductive-age women. Data were collected with pre-tested, structured, interviewer-administered questionnaires. Data were coded and entered into Epi info version 7 and exported to SPSS version 20. Bivariable and multivariable logistic regression models were applied. A P-value0.05 was considered to declare a result as significant at 95% CI.

**Result:**

The overall unmet need in this study area was 22.6%, from whom 15.1% of respondents were wanted children later and 7.5% were wanted no more children. For women who had been visited by health care providers within 12 months before the study, women currently on menstrual status, the desired number of children, and induced abortion were found statistically significant.

**Conclusion:**

The unmet need for FP was found high in the study area as compared to the national and regional prevalence. Women visited by health care providers, currently menstruating, the desired number of children, and history of induced abortion were significantly associated with the unmet need of modern FP. Health care providers and health extension workers need to visit regularly and promote appropriate and active IEC programs that address the provision of accurate information about the availability of the services and various contraceptive options including techniques to reduce and change perceived barriers to service utilization (such as rumors and misconceptions of FP).

## Background

Women who are sexually active and want to avoid becoming pregnant but are not using contraception are called to have an unmet need for family planning. The unmet need of family planning has two divisions. These are unmet needs for spacing or want later and unmet needs for limiting [1].

About 74 million unintended pregnancies are reported each year in developing countries. Most of them are women using no contraception or using traditional methods. There would be a chance to avoid 52 million unmet needs and 70,000 women from pregnancy-related deaths if all unmet needs were met [1]. Currently, in the world, the unmet need for modern family planning is still not low especially in developing countries there is a greater prevalence of unmet need for family planning [2]. In 2014 USAID reported the unmet need of modern family planning among young women is different in different regions of the world. West and Central Africa which is 29.3% in young women and 25.9% in older women, East and South Africa 25.5% in younger women and 25% in older women, Latin America and Caribbean 24.5% in younger women and 15.6% in older women and the Middle East and North Africa 10.8% in younger women and 12.6% in older women [3–5].

Ethiopia is densely populated with its rapidly increasing population. The use of modern family planning methods especially that of long-term FP is still low [6]. Despite efforts made to improve access and use of family planning nationally, the unmet need for FP is still not reduced as needed [7]. According to the Ethiopian Demographic and Health Survey 2016, the national prevalence of unmet needs for family planning is 22% [3].

The unmet need for family planning becomes one of the reasons for unwanted pregnancy, especially in developing countries like Ethiopia [8, 9]. If unmet needs become decreased, the Contraceptive prevalence rate will increase from 36% to 55% among women of the country [3, 10, 11].

In the context of the Ethiopian situation, even though there are studies on prevalence and factors of modern family planning among women, still there is a higher magnitude of unmet need of family planning even under the influence of underreporting when it is seen according to Ethiopian 2020 family planning plan which is 10% [2]. Currently, there are some important reasons for the unmet need of modern family planning such as low performance of health staff on family planning services especially on counseling about the importance of modern methods compared to traditional methods, telling about side effects clearly and effectively, addressing client questions, manage side effects and complications effectively, allow free choice of family planning methods and detail discussion on misconceptions raised in the community [12–14].

So, this study was relevant to look into the problem and gives more information about the prevalence and associated factors of unmet need of modern family planning among women in the South Gondar Zonal Administration.

## Methods of the study

### Study design and setting

A Community based cross-sectional study design was conducted from January 09-23/2017 in the South Gondar Zone. South Gondar is one of the 11 zonal administrations in Amhara regional state, Ethiopia. It has 13 woredas, 32 urban, and 138 rural kebeles. There are 12 primary hospitals, 73 health centers, and 145 health posts. All health facilities provide family planning services. It has 2,039,077 total populations (Health department’s report of 2016).

### Population

All reproductive-age women found in the south Gondar zone were used as the source population of the study. Reproductive age women who lived in selected kebeles during the data collection period were the study population of this study.

### Inclusion and exclusion criteria

All reproductive age group women were included in the study. But reproductive-age women who were severely ill and who lived less than 6 months in the selected kebeles were excluded.

### Sample size determination and sampling procedure

Sample size was determined using a single population proportion formula based on the following assumptions: prevalence of unmet need of modern family planning 22% [3]; degree of margin 5%; 1.5 design effect and 10% non-response rate at 5% level of significance:

n = (Za/2)^2^ p (1-p) /d^2^

= (1.96)^2^ × (0.22) × (0.78) / (0.05)2 = 263

= 263×1.5 (design effect) = 396 by adding 10% non-response rate = 396 +40 = 436

Sample size using double population proportion was determined for three variables from previous studies and the final results. The final sample size used was 528 reproductive-age women (Table 1).

**Table 1:** Sample size calculation with double population proportion formula using Epi-info version 7 for the unmet need of modern FP in South Gondar, Ethiopia, 2017 [7, 9, 15].

Factors	%outcome exposed	Power	% outcome unexposed	OR	10%	Design effect	Sample size
Partner disapproval	11	80%	41	2.63	32	1.5	528
Have you ever used FP? No	43.7		56.3	2.29	23		379
Number of living children(3/4)	32.2		67.8	2.84	20		324

There were 170 kebeles in south Gondar. The sampling technique applied was multistage sampling [5 woredas (Ebnat, Farta, Fogera, Tach-gaynt & Hamusit), 12 kebeles, and 528 households]. By simple random sampling (lottery method), 5 woredas and 12 kebeles were selected within the selected woredas considering the feasibility of this study. Proportional to size allocation of samples for each kebeles was conducted after the number of reproductive-age women were taken from a family folder of respective health extension workers at kebele. Then, to access each woman in each kebeles, a systematic sampling technique, by the use of household numbers, was applied. Finally, reproductive-age women in the selected households were interviewed. When there was more than one woman in a household, one of them the woman was selected by lottery method for interview. If sampled women were not found or if their homes were closed, then data collectors were returned for three rounds and if accessed she was interviewed.

### Variables of the study

Unmet need for family planning was the dependent variable, and the independent Variables were: Socio-demographic variables like age, residence, religion, educational background, education of husband, occupation of the husband; reproductive factors: perceived risk of pregnancy, parity, number of children, desire number of children, menstrual status, unintended pregnancy and induced abortion; family planning factors: awareness on FP, place to access FP methods, a side effect of FP methods, shortage of FP methods, and fear of infertility; health facility and Health provider’s related factors: distance of health facility, waiting time, the information given, appointment, free choice of FP methods; religious, cultural and traditional related factors: roles of religious leaders, religious desire to space, cultural factors, traditional factors, opposition from community leaders and fear of infidelity [15–18].

### Operational definition

Unmet need for FP: women who are not pregnant and not in postpartum amenorrhea and are considered fecund and want to postpone their next birth for 2 or more years or stop childbearing altogether but are not using a contraceptive method or have a mistimed or unwanted current pregnancy, or are postpartum amenorrhea and their last birth in the last 2 years was mistimed or unwanted [12, 15, 19, 20]. So, the unmet need of FP is who does not want any more children but does not use any FP method [3, 21]. The dependent variable was an unmet need for family planning and was dichotomized as having unmet need (1 = yes) and absence of unmet need (0 = no).


$$Unmet\;need\;for\;family\;planning=\frac{\begin{array}{c} Women\;of\;reproductive\;age\;(15-49)who\;are\;married\;or\;in\;a\\ \;union\;and\;who\;have\;an\;unmet\;need\;for\;family\;planning \end{array}}{\begin{array}{c} \;a\;total\;number\;of\;women\;of\;reproductive\;age\;(15-49)\\ who\;are\;married\;or\;in\;a\;union \end{array}}*100$$

### Data collection tools, procedures, and data quality control

Data collection tools were adopted from roughly reviewing works of literature [3, 7, 12, 15, 22–24]. A pretested structured interviewer-administered questionnaire was used and face-to-face interviews were conducted to collect data. Data were collected by three BSc nurses after training for one day was given. In addition to the principal investigators, one experienced BSc nurse supervisor was assigned to lead the data collection, check for completeness and consistency of questioners, and to assist data collectors. If respondents did not exist, data collectors returned in next day and interviewed them again.

The pre-test was done on 26 (5%) respondents in the Makisegnit district, north Gondar before actual data collection. Supervision and on-the-spot checking of the data collection procedures were made by the assigned supervisor and principal investigator. Every day, at the end of the data collection process, the discussion was conducted with the supervisor and data collectors, and problems encountered were identified and timely addressed. The completeness of the questionnaire was checked before data entry.

### Data processing and analysis

After the data collection, it was checked for completeness and entered into Epi-info Version 7, and exported to SPSS Version 20 statistical software for statistical analysis. Descriptive analyses like frequency distribution, mean and standard deviation were computed to summarize data. To assess the association between the unmet need of family planning and independent variables, a bivariable analysis was performed. Then, variables with a p-value less than 0.25 were fitted into the multivariable logistic regression model to identify statistically significantly associated variables with unmet needs of modern FP using p-value ≤0.05, and adjusted odds ratio (AOR) at 95% CI were used to determine predictors of unmet need of family planning.

### Ethical consideration

Ethical approval and clearance were taken from the institutional review board (IRB) of the University of Gondar, College of Medicine and Health Sciences. Letter of permission to conduct the study was obtained from the administrative office of the South Gondar Zone. Written informed consent was obtained from participants and/or parents (guardians) of minor participants before data collection. They were informed that participating in the study was voluntary. The right to withdraw from the study at any moment during the interview was assured. Teenagers (≥ 15 years old) were interviewed with their families to support the interview process. Confidentially of information was secured to keep their privacy.

## Result

### Socio-demographic characteristics

A total of 483 women have participated in the study with a response rate of 91.4%. Two hundred fifteen (44.5%) of reproductive age group women were aged 25–34 years with the mean age of 32 (SD ± 7.916). The youngest and the oldest age were 15 and 49 years respectively. Of 483 respondents, 112 (23.2%) were living in an urban area (Table 2).

**Table 2:** Socio-demographic characteristics of reproductive age women in South Gondar, Ethiopia, 2017 (n = 483).

Variables	Category	Frequency	Percent
Age	15–24	113	23.4
	25–34	215	44.5
	> = 35	155	32.1
Residence	Urban	112	23.2
	Rural	371	76.8
Religion	Muslim	297	61.5
	Orthodox	174	36.0
	Others*	12	2.5
Educational status of respondents	no formal education	162	33.5
	Primary	265	54.9
	secondary and above	56	11.6

• Others-protestant and catholic.

### Family planning related factors

Most of the women 464 (96%) were heard about modern FP from different sources. Moreover, 374 (77%) of respondents were current FP users. One hundred nine (23%) of women were not using modern FP due to different reasons (Table 3).

**Table 3:** Family planning-related factors in South Gondar, Ethiopia, 2017 (n = 483).

Variables	Category	Frequency	percent
Have you heard about modern FP	Yes	464	96.1
	No	19	3.9
If yes where do you get the information	Relatives and friends	204	44.0
	Health professions	154	33.2
	Health professionals, relatives, and friends	47	10.1
	Husband	28	6.0
	Media	17	3.7
	Local news and magazines	14	3.0
Are you currently using modern FP?	Yes	374	77.4
	No	109	22
If yes from where?	Health center	209	55.9
	Health post	89	23.8
	Private clinic	37	9.9
	Hospital	26	7
	Drug store	13	3.5
If no reason	No awareness	30	27.5
	Pregnant	26	23.9
	Husband influence	19	17.4
	FP side effect	17	15.6
	Fear of infertility	9	8.3
	Use of traditional methods	4	3.7
	Religion	4	3.7

### Health facility and health workers related factors

Of all respondents, 320(66%) were visited by health providers. The time taken for the round trip to visit health institution were < 60 minutes long for 329 (68%) respondents. In addition, 110 (23%) of the respondents were spent their time in health institutions for 30 minutes. Out of all 357 (74%) of the respondents were told about FP options. An appointment had given for 332 (88%) of the FP users (Table 4).

**Table 4:** Health facility and health workers related factors for unmet needs of modern family planning methods in South Gondar, Ethiopia, 2017 (n = 483).

Variables	Category	Frequency	Percent
Have you been visited by an FP provider in the last12 months?	Yes	320	66.3
	No	163	33.7
Time is taken for round trip	0–60 minutes	329	68.1
	>60 minutes	154	31.9
Time is taken in a health facility	0–30 minutes	110	22.8
	>30 minutes	372	77.2
Have you been told about FP options?	Yes	357	74
	No	126	26
If yes, do you get the method you want?	Yes	265	74.2
	No	92	25.8
Do you get an appointment?	Yes	332	87.8
	No	46	12.2

### Religious, cultural, and traditional related factors

Most of the respondents 432 (89%) of reproductive age women accept modern FP. Seventy-six (16%) of the respondents get opposition from religious leaders for FP utilization. One hundred seventy-five (36%) of respondents reported that there were traditional views in their community of these views 49 (28%) said modern FP reduces body weight, 47 (27%) reported that it results in difficulty to work with the hand, 43(24.6%) of them said it causes menses to retain in the uterus and 36(20.6%) reported as it causes infertility (Table 5).

**Table 5:** Religious, cultural, and traditional related factors in South Gondar, Ethiopia, 2017 (n = 483).

Variables		Frequency	Percent
Is modern FP accepted in your religion?	Yes	432	89.4
	No	51	10.6
Opposition from religious leaders	Yes	76	15.7
	No	407	84.3
Opposition from community leaders	Yes	42	8.7
	No	441	91.3
Are there traditional views on modern FP?	Yes	175	36.2
	No	308	63.8
If yes, what are they?	losses body weight	49	28.0
	difficult to work with hand	47	26.9
	menses retain in the uterus	43	24.6
	causes infertility	36	20.6

### Reproductive related factors characteristics

Four hundred forty-seven (92.5%) of respondents were currently menstruating. Out of all 449 (93%) of respondents had ever been pregnant. Four hundred thirty-five (90%) of the respondents gave birth. Two hundred ninety-four (61%) of women desired to have <5 children. Eighty-five (17.6%) of respondents had a history of unwanted pregnancy due to 56 (65.9%) of them did not use modern FP methods. Only 34 (7%) of the respondents had a history of induced abortion due to 17 (89.5%) of them not use modern FP methods (Table 6).

**Table 6:** Reproductive-related factors characteristics of reproductive age group women in South Gondar, Ethiopia, 2017 (n = 483).

Variables	Category	Frequency	percent
Are you currently menstruating	Yes	429	89.5
	No	54	10.5
Have you ever been pregnant	No	34	7.0
	Yes	449	93.0
give birth	No	48	9.9
	Yes	435	90.1
desire number of children	0–5	294	60.9
	>5	189	39.1
total alive children	0–5	452	93.6
	>5	31	6.4
History of unintended pregnancy	No	398	82.4
	Yes	85	17.6
if yes reason	do not use modern FP	56	65.9
	mistimed use of modern FP	26	30.6
	failure of modern FP	3	3.5
Have your history of induced abortion	No	449	93.0
	Yes	34	7.0
if yes reason	do not use modern FP	17	89.5
	failure of modern FP	2	10.5

### Unmet need of modern family planning

The overall unmet need of modern family planning in the study area was 22.6% at 95% CI: (18.8% - 26.5%).

### Factors associated with the unmet need of modern FP

The results of the bivariable and multivariable logistic regression model showed that reproductive-aged women visited by health care providers, currently menstruating, having the desired number of children, and induced abortion were found statistically significantly associated with unmet needs of modern FP.

Women who had not been visited by health care providers within 12 months before the study period were 0.34 times less likely to have an unmet need of modern FP compared to women who had been visited (AOR = 0.34, 95% CI: (0.21–0.54)). Women who do not currently menstruate were 3.75 times more likely to have an unmet need for FP (AOR = 3.75, 95% CI: (1.48–13.0)) and those women who had desired to have >5 children were 0.47 times less likely to have an unmet need for FP (AOR = 0.47, 95% CI: (0.38–0.74)). Finally, women who had no induced abortion history were 1.3 times more likely to have an unmet need of modern family planning methods (AOR 1.30 95% CI: (1.01–7.91)) (Table 7).

**Table 7:** Factors associated with the unmet need of modern family planning among reproductive-aged women in South Gondar, Ethiopia, 2017 (n = 483).

Explanatory variables	Unmet need		COR (95%CI)	AOR(95%CI)
	Yes	No		
Residence				
Urban	32	80	1	1
Rural	77	294	0.65 (0.36–0.94)	0.43(0.29–0.61)
Visited by providers				
Yes	55	108	1	1
No	54	266	0.39(0.26–0.62)	0.34(0.21–0.54) _*_
Currently Menstruating				
Yes	90	339	1	1
No	19	35	2.04(1.51–10.35)	3.75(1.48–13) _*_
Desire no children				
0–5	82	212	1	1
>5	27	162	0.43(0.27–0.70)	0.47(0.38–0.74) _*_
History of Induced abortion				
Yes	14	20	1	1
No	105	344	2.29(1.79–6.65)	1.30(1.91–7.91) _*_
Give birth				
Yes	103	332	2.17(1.90–5.25)	3.23(2.1–5.7)
No	12	36	1	1

_*_ p—Value < 0.05.

## Discussion

This study tried to assess the prevalence of unmet needs of modern family planning among reproductive-aged women in South Gondar, Ethiopia. The proportion of unmet need was found to be 22.6% at 95% CI: (18.8–26.5). Of this, 15.1% of total unmet need was an unmet need for spacing and 7.5% of total unmet need was an unmet need for limiting. This finding was in line with the prevalence of unmet need in Africa (22%), Ethiopian national prevalence of unmet need (22%) (13% for spacing and 9% for limiting), and a study conducted in the Tigray region, Ethiopia 21.4% (14.5% for spacing and 6.9% for limiting) [3, 9, 25, 26] respectively.

On the other side, this finding was higher than the world unmet need of family planning in 2017 (12%), unmet need in Oceania (15%), North America (10%), Latin America (10%), Asia (10%), and Caribbean (9%) [2]. These variations may be due to differences in the level of awareness of study participants. Other possible explanations for the observed variations could be research design, culture, and population differences.

The result of this study revealed a significant association between women not visited by health providers towards the unmet need of modern FP. The odds of unmet need of modern FP among reproductive age group women who did not visit by health care providers were 0.34 times lower than women who were visited by health providers within the last 12 months before this study period. This finding was different in the direction from the previous study done in Enemay woreda, North West Ethiopia [15]. Those who had not visited were more likely to have unmet needs than visited. This variation may be due to research design.

The result also showed the presence of a significant association between the desired number of children and the unmet need of modern family planning. The probabilities of women who had the desire of > 5 children were 0.47 times lower than women who had desired 0–5 children. Even if people need different family sizes, the number of children increases, the probability of using modern family planning will also increase. This study is in contrast with a study done in Tigray [9]. This difference may be due to differences in awareness levels on the importance of planning family size.

Women who did not menstruate had 3.75 times more likely to have unmet needs than menstruating women. This study is in line with the study done in Enemay woreda [15]. This may be due to similarities in study design.

Women who had no history of induced abortion had 1.3 times higher unmet need of modern family planning than those who had induced abortion history. Because women with a history of induced abortion have more awareness in reducing unmet needs than others. This study is congruent with the study done by Kelemu Chafo and Feleke Doyore [26]. This similarity would be due to the study design and sampling procedure.

### Limitation of the study

Limitations of the study given the great role men play in decision making concerning unmet needs, this research has a weakness that it did not evaluate unmet needs among men. In addition, our definition for unmet need was not directly applicable to sexually active women out of union given that other parameters are to be taken into consideration. Our study did not evaluate the supply and delivery of the family planning services which could be possible factors influencing unmet needs.

## Conclusions

The unmet need for FP was found high in the study area as compared to the national and regional prevalence. Women visited by health care providers, currently menstruating, the desired number of children, and history of induced abortion were significantly associated with the unmet need of modern FP. Health care providers and health extension workers need to visit regularly and promote appropriate and active IEC programs that address the provision of accurate information about the availability of the services and various contraceptive options including techniques to reduce and change perceived barriers to service utilization (such as rumors and misconceptions of FP).
